# A simple prediction model of hyperuricemia for use in a rural setting

**DOI:** 10.1038/s41598-021-02716-y

**Published:** 2021-12-02

**Authors:** Jia-Cheng Shi, Xiao-Huan Chen, Qiong Yang, Cai-Mei Wang, Qian Huang, Yan-Ming Shen, Jian Yu

**Affiliations:** 1 Department of Nephrology, Haining People’s Hospital, No. 2 West Qianjiang Road, Jiaxing, 314400 Zhejiang China; 2 Department of Endocrinology and Rheumatology, The First People’s Hospital of Linping District, No. 369 Yingbin Road, Hangzhou, 311100 Zhejiang China; 3 Department of Endocrinology, The Affiliated Hospital of Guilin Medical University, No. 15 Lequn Road, Guilin, 541001 Guangxi China; 4 Department of Laboratory Medicine, The Affiliated Hospital of Guilin Medical University, No. 15 Lequn Road, Guilin, 541001 Guangxi China

**Keywords:** Endocrine system and metabolic diseases, Metabolic disorders

## Abstract

Currently, the most widely used screening methods for hyperuricemia (HUA) involves invasive laboratory tests, which are lacking in many rural hospitals in China. This study explored the use of non-invasive physical examinations to construct a simple prediction model for HUA, in order to reduce the economic burden and invasive operations such as blood sampling, and provide some help for the health management of people in poor areas with backward medical resources. Data of 9252 adults from April to June 2017 in the Affiliated Hospital of Guilin Medical College were collected and divided randomly into a training set (n = 6364) and a validation set (n = 2888) at a ratio of 7:3. In the training set, non-invasive physical examination indicators of age, gender, body mass index (BMI) and prevalence of hypertension were included for logistic regression analysis, and a nomogram model was established. The classification and regression tree (CART) algorithm of the decision tree model was used to build a classification tree model. Receiver operating characteristic (ROC) curve, calibration curve and decision curve analyses (DCA) were used to test the distinction, accuracy and clinical applicability of the two models. The results showed age, gender, BMI and prevalence of hypertension were all related to the occurrence of HUA. The area under the ROC curve (AUC) of the nomogram model was 0.806 and 0.791 in training set and validation set, respectively. The AUC of the classification tree model was 0.802 and 0.794 in the two sets, respectively, but were not statistically different. The calibration curves and DCAs of the two models performed well on accuracy and clinical practicality, which suggested these models may be suitable to predict HUA for rural setting.

## Introduction

Hyperuricemia (HUA) is a chronic metabolic disease caused by purine metabolic disorders, which can result from congenital purine metabolic abnormalities, systemic diseases or the use of drugs such as anti-tubercular drugs, diuretics and immunosuppressant agents^1^. Its clinical features include fluctuating or persistent increases in blood uric acid, gout and interstitial nephritis. In severe cases, joint deformities or uric acid urinary tract stones may occur^2^. With the continued improvement of China's economic level, the prevalence of diseases such as HUA is on the rise. A population study based on big data showed that from 2014 to 2017, the prevalence rate of HUA in Chinese adults increased from 18.29 to 23.06%^3^. At present, there were many studies to show that HUA is positively correlated with hypertension^4^, diabetes^5^, metabolic syndrome^6^, acute myocardial infarction^7^, coronary heart disease^8^, acute kidney injury^9^, chronic kidney disease^10^, high incidence of cancer and mortality^11^. However, there is still a lack of evidence on whether lowering uric acid can improve the prognosis of related diseases^12,13^. It is worth noting that there are many views advocating no intervention for asymptomatic HUA^14,15^, but usually Chinese and Japanese doctors are more active in the treatment of this condition, including health guidance on diet and exercise, and the popularization of HUA-related common sense^16,17^. In general, the popularization of HUA in China is not sufficient, and the patients' awareness rate and cure rate of HUA are relatively low. Therefore, early detection and prevention of HUA may be helpful to improve the quality of people's life and reduce the social burden.

In previous studies, genetic tests^18^ and dietary information^19^ were used in an attempt to predict the risk of HUA, but at present, these criteria have still been widely used to verify the incidence and risk factors of HUA by recording lifestyle characteristics and blood biochemical indicators^20^. Taking into account the large population base in China, the large proportion of the rural inhabitants, the imbalance of medical resources and the lack of corresponding facilities and equipment in many rural hospitals, coupled with the need to reduce the financial burden and avoid invasive procedures such as taking blood, we attempted to predict the risk of HUA based on some basic information of the patients and non-invasive examinations, including age, gender, body mass index (BMI) and the prevalence of hypertension.

Logistic regression is often used to look for risk factors associated with disease as well as to predict the probability of the occurrence of certain diseases. It is a specific and simple traditional prediction method, and it can visually present the results pictorially through the nomogram model. However, when dealing with complex relationships between multiple factors, the errors involved in analysis will tend to increase. The decision tree model is a simple and easy-to-use non-parametric classifier, among which the classification and regression tree (CART) algorithm is the most widely used algorithm for this model. When the dependent variable is the classification variable, the generated decision tree is the classification tree. The classification tree model is a tool to represent processing logic by using a binary tree diagram, which has no strict restrictions on the type of data and can make up for the shortcomings of traditional statistical methods^21^. Therefore, in this study, the nomogram and the classification tree models were combined to fully explore the physical examination database and construct a HUA prediction model suitable for people living in rural settings. It was anticipated that this procedure would produce a high accuracy, low cost and easy to use model which can provide assistance for the health management of rural community populations by helping in the prevention of HUA.

## Materials and methods

### Study participants

The study population for this cross-sectional survey came from adults who underwent physical examinations at the Physical Examination Center of the Affiliated Hospital of Guilin Medical College from April to June 2017. All participants underwent questionnaire surveys as well as physical and laboratory examinations and patients with severe renal impairment (estimated glomerular filtration rate < 15 ml/min/1.73 m^2^), severe liver impairment (alanine aminotransferase or aspartate aminotransferase > 120 U/L), malignant tumors and pregnant women were excluded. Finally, a total of 9252 adults aged 18–90 years old were included in the study which was approved by the Ethics Committee of Guilin Medical College (approval number: GLMUIA2019064), and all participants gave written informed consent.

### Data selection and measurements

We adopted a questionnaire survey method to collect the general information and clinical data of the research population, including: (1) basic personal information (name, gender and age); (2) previous medical history (hypertension, diabetes, chronic kidney disease stage 5 with regular dialysis treatment, and tumor history) and (3) pregnancy history. The height, weight and blood pressure of the subjects were measured as part of the physical examination. The height was measured without a crown or shoes while standing upright (the value obtained was accurate to 0.01 m). The weight was measured without shoes and an empty bladder (the value obtained was accurate to 0.1 kg), and the BMI was calculated (BMI = weight (kg)/height^2^ (m^2^)). Before taking the blood pressure measurements, subjects were required to avoid drinking alcohol, smoking, drinking tea, coffee or performing vigorous exercise and resting for at least 5 min. An Omron HBP-9020 automatic medical blood pressure meter was used for measurements on all subjects. They were required to be in a sitting position and the blood pressure was measured from the right brachial artery twice at an interval of 1–2 min and the average value taken. If there was a big difference between the two measurements, the blood pressure of the right brachial artery was measured for a third time, and the results were averaged for the three readings, with the value obtained being accurate to within 1 mmHg. During laboratory examination, subjects were asked to eat a low-purine diet at dinner the previous day with details of what they should not indulge in being given to them. The venous blood was drawn after the subjects had an empty stomach for more than 10 h in order to detect serum uric acid (SUA), serum creatinine (SCr), blood urea nitrogen (BUN), alanine aminotransferase (ALT) and aspartate aminotransferase (AST) levels. The Modification of Diet in Renal Disease (MDRD) equations was used to calculate the estimated glomerular filtration rate (eGFR)^22^, renal insufficiency was defined as eGFR < 90 ml/min/1.73 m^2^. All measurements were performed by well-trained doctors, nurses and researchers.

The diagnostic criteria of hypertension used was in accordance with the diagnostic criteria of the "2018 China Guidelines for the Prevention and Treatment of Hypertension"^23^: systolic blood pressure ≥ 140 mmHg and/or diastolic blood pressure ≥ 90 mmHg and/or a previous history of hypertension diagnosed as hypertension. The diagnosis of HUA was as stated in the "Guidelines for the Diagnosis and Treatment of Hyperuricemia and Gout in China (2019)"^24^ and HUA was diagnosed when the SUA level was higher than 420 μmol/L for both men and women.

### Statistical analysis

SPSS 25.0 (IBM, USA), STATA (version 15.1) and R software (version 4.0.0) with the rms package were used for data processing and statistical analyses. The counts data were represented by *n* (%), and chi-squared test was used for comparison between groups. Measurement data were represented by $\overline{x}$ ± *SD*, and comparisons between groups were performed by Student’s *t*-test. The model construction and analysis can be divided into three stages:

In the first stage, the method of pure random sampling was used to select 70% of the sample size to form a training set for calculating parameters and building the model, and the remaining 30% of the sample size was used to form a validation set for testing and evaluating the model.

In the second stage, two models were constructed. The establishment of a nomogram model: for the training set data, univariate logistic regression analysis was carried out first. Variables with *P* < 0.05 were further analyzed by multivariate logistic regression analysis. On this basis, the rms package, which offers a variety of tools to build and evaluate regression models in R software, was used to establish a nomogram which could directly display the model. For establishment of the classification tree model, SPSS 25.0 was used for analysis and modeling in the training set, and debugging of the important parameters such maximum tree depth and minimum number of cases. After verification of the training set, when the maximum tree depth = 4, parent node = 200 and child node = 100, the model was deemed to have reached the optimal level.

The third stage was the evaluation of the models. The receiver operating characteristic (ROC) curves were drawn to analyze the sensitivity and specificity of the two models, and the discriminability of the two models was evaluated by comparing the area under ROC (AUC). Using the rms package and drawing a calibration curve, the accuracy of the prediction of the two models was further evaluated. Finally, in order to determine whether the two models could improve patient prognosis, decision curve analysis (DCA) was drawn to evaluate the clinical practicality of the two models. All statistical tests were two-sided tests, and *P* < 0.05 was considered as statistically significant.

### Ethical approval

This study was approved by Ethical Committee of Guilin Medical University in China (approval number: GLMUIA2019064). All research was performed in accordance with relevant guidelines. Informed consent was obtained from all participants and/or their legal guardians.

## Results

### Clinical characteristics of the subjects

The clinical characteristics of the population in this study are shown in Table 1. In this study, 2023 HUA patients (1861 males and 162 females) were identified, with an average SUA level of 482.41 ± 52.00 μmol/L, and 7229 non-HUA patients (3471 males and 3758 females), with an average SUA level of 312.52 ± 61.55 μmol/L. There were 6364 and 2888 cases in the training and validation sets, respectively.

**Table 1:** Clinical characteristics of the population used in this study.

Variable	Training set (*n* = 6364)		*P* value	Validation set (*n* = 2888)		*P* value
	Non-HUA (*n* = 4960)	HUA (*n* = 1404)		Non-HUA (*n* = 2269)	HUA (*n* = 619)	
Age (years)	42.71 ± 12.59	44.30 ± 13.17	< 0.001	42.75 ± 12.79	44.11 ± 13.36	0.021
**Gender**			< 0.001			
Male	2399	1295		1072	566	
Female	2561	109		1197	53	
BMI (kg/m^2^)	23.65 ± 3.29	26.25 ± 3.35	< 0.001	23.58 ± 3.17	25.98 ± 3.38	< 0.001
**Hypertension**			< 0.001			< 0.001
No	4064	954		1848	423	
Yes	896	450		421	196	
SUA (μmol/L)	312.21 ± 61.50	481.98 ± 50.87	< 0.001	313.19 ± 61.67	483.37 ± 54.49	< 0.001
SCr (μmol/L)	75.82 ± 16.76	76.63 ± 15.93	0.106	76.03 ± 16.50	76.76 ± 18.07	0.338
BUN (mmol/L)	4.46 ± 1.22	4.44 ± 1.13	0.531	4.46 ± 1.20	4.52 ± 1.27	0.311
ALT(U/L)	20.00 ± 13.26	29.56 ± 17.85	< 0.001	19.66 ± 12.62	28.52 ± 15.89	< 0.001
AST(U/L)	19.13 ± 6.94	22.87 ± 8.96	< 0.001	19.01 ± 6.87	22.26 ± 7.89	< 0.001
**Renal insufficiency**			< 0.001			< 0.001
No	2987	1006		1355	441	
Yes	1973	398		914	178	
**Diabetes**						
No	4711	1316	0.065	2148	589	0.630
Yes	249	88		121	30	

Participants in the training and validation sets had similar clinical characteristics, with no statistical difference in age, gender, BMI, prevalence of hypertension, prevalence of SUA, SCr, BUN, ALT, AST, prevalence of renal insufficiency and prevalence of diabetes (*P* > 0.05). In the training set, the age, gender, BMI, prevalence of hypertension, ALT, AST and prevalence of renal insufficiency of HUA patients were statistically different from those of non-HUA patients (*P* < 0.05). The comparison of clinical characteristics between the two groups in the validation set was consistent with the training set (Table 1).

### Establishment and exhibition of nomogram model

**Figure 1:**
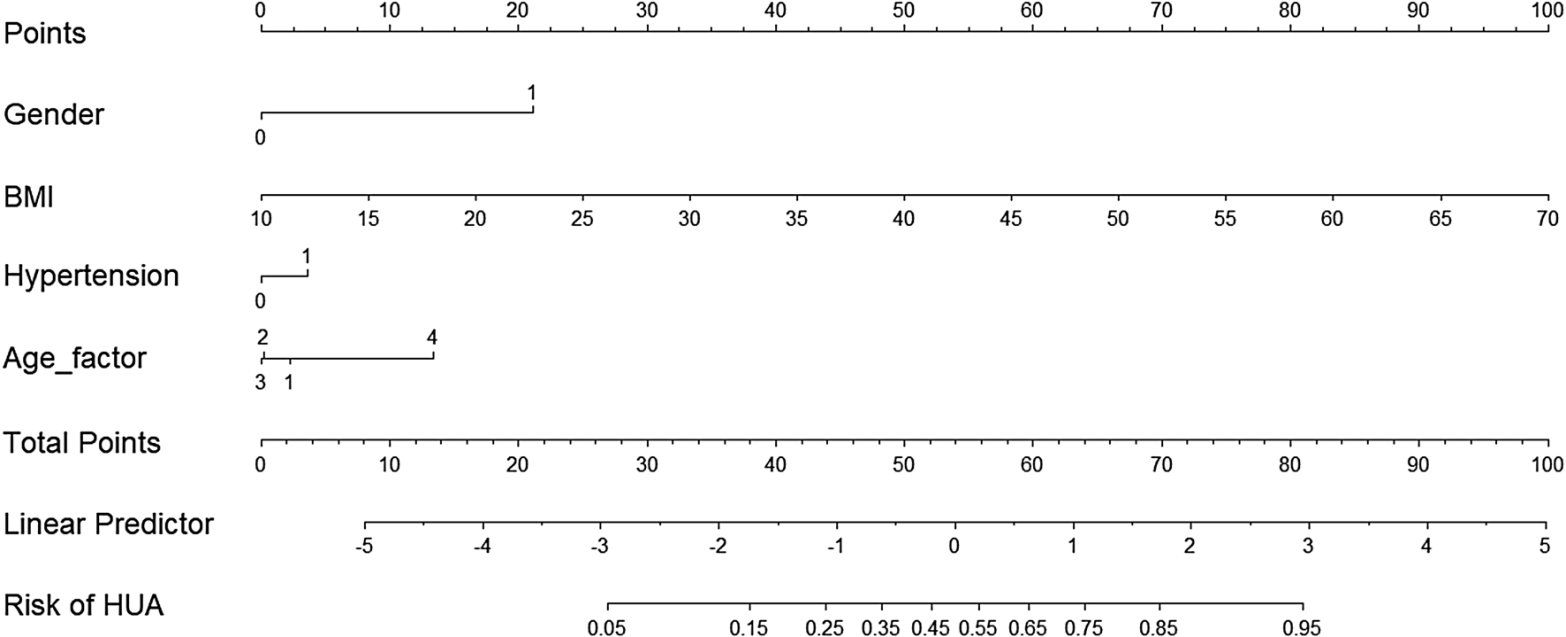
The usage method of nomogram to predict the risk of HUA. The scores were calculated according to the following factors (gender, BMI, hypertension and age) of the enrolled patients, and sum the total scores was used to calculate the risk of HUA. The figure was created by using R software (version 4.0.0), URL: https://www.r-project.org/.

The basic information and the non-invasive examination of the included population including age, gender, BMI and hypertension were subjected to single factor logistic regression analysis. The results showed that the risk factors of HUA included the above four indicators, and the results were statistically significant (*P* < 0.05). Age (assigned to four groups: 18–29 years old = 1, 30–49 years old = 2, 50–69 years old = 3, ≥ 70 years old = 4), gender (assigned to two groups: female = 0, male = 1), hypertension (assigned to two groups: no = 0, yes = 1) and BMI were taken as independent variables, and hyperuricemia (assigned to two groups: no = 0, yes = 1) as a dependent variable, and then further multivariate unconditional logistic regression analysis was performed. The results suggest that old age (OR = 3.382, 95%CI 1.707–6.701), male (OR = 10.039, 95%CI 8.146–12.372), high BMI (OR = 1.199, 95%CI 1.174–1.225) and hypertension (OR = 1.478, 95%CI 1.260–1.733) were independent risk factors for HUA (Table 2). Based on these results, a nomogram model was established by using R4.0.0 and a rms package to predict HUA occurrence (Fig. 1).

**Table 2:** The results of multivariate logistic regression analysis on the occurrence of hyperuricemia.

Variables	*β*	SE	Ward value	*P* value	*OR*	95%*CI*
Gender	2.307	0.107	468.163	0.000	10.039	8.146 ~ 12.372
**Age**						
18 ~ 29			23.267	0.000	1	Reference
30 ~ 49	-0.216	0.096	5.026	0.025	0.805	0.667 ~ 0.973
50 ~ 69	-0.242	0.104	5.378	0.020	0.785	0.640 ~ 0.963
≥ 70	1.219	0.349	12.202	0.000	3.382	1.707 ~ 6.701
Hypertension	0.390	0.081	23.083	0.000	1.478	1.260 ~ 1.733
BMI	0.182	0.011	274.227	0.000	1.199	1.174 ~ 1.225

### Establishment of the classification tree model

**Figure 2:**
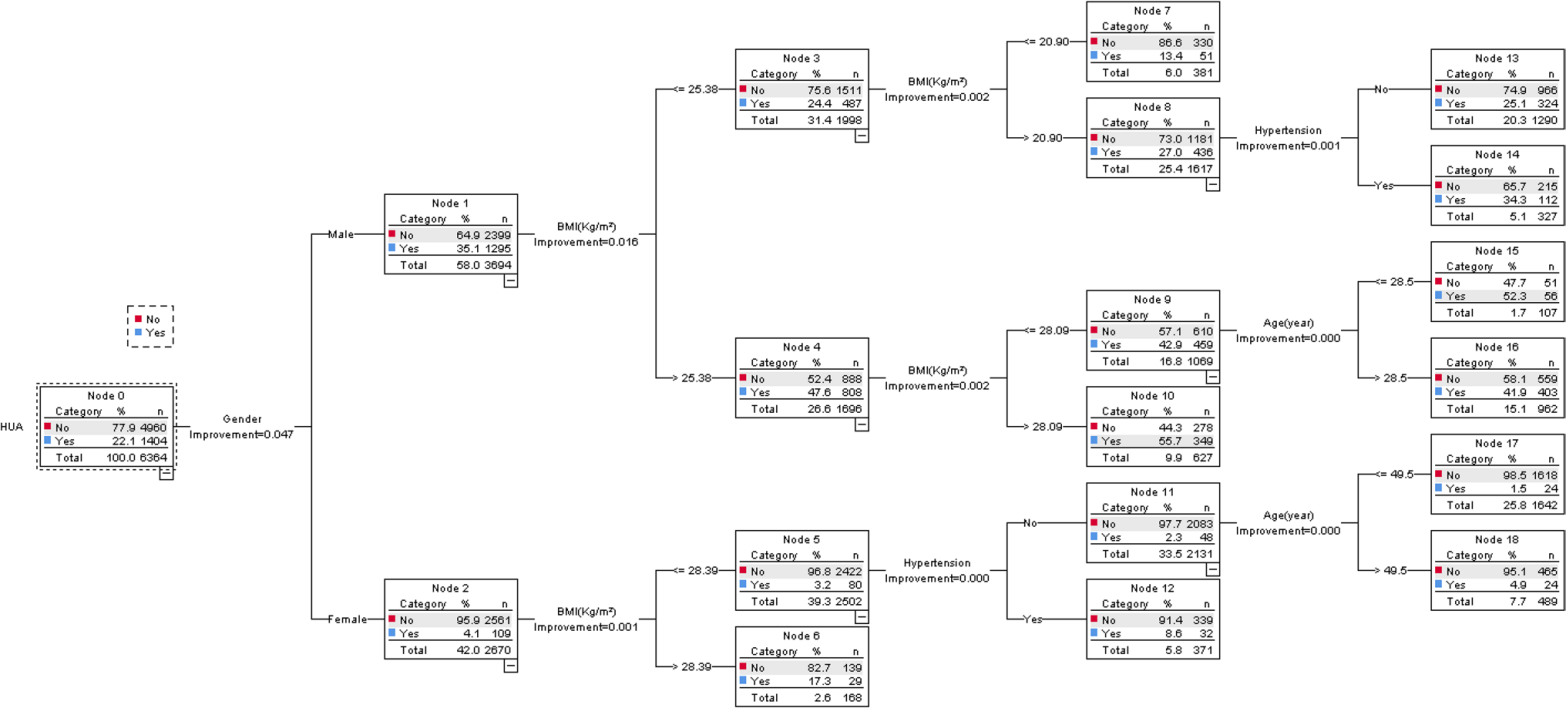
The classification tree model of HUA patients. The figure was created by using SPSS 25.0 (IBM, USA), URL: https://www.ibm.com/products/spss-statistics/. HUA: hyperuricemia; BMI: body mass index.

Age, gender, BMI and hypertension were taken as influencing factors of HUA patients and used to construct a classification tree model. Based on the growth and pruning of the minimum sample sizes of root and child nodes, the classification tree model was concluded to include 4 layers, 18 ordinary nodes and 6 terminal nodes (Fig. 2). The results of the classification tree model showed that the importance of HUA factors ranged from high to low in terms of gender, BMI, hypertension and age, respectively.

### Validation and evaluation of the model

**Figure 3:**
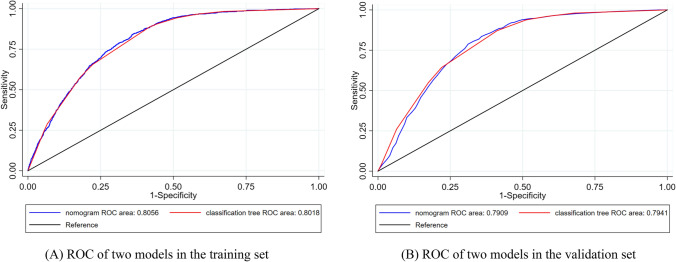
ROC curves of the two models in the training and validation sets. The figure was created by using STATA (version 15.1), URL: https://www.stata.com/.

The ROC curve was used to evaluate the discriminability of the two models. The AUC of the nomogram model constructed in this study was 0.806 and 0.791, the specificity was 63.83% and 64.20% and the sensitivity was 84.40% and 83.52% in the training and verification sets, respectively. The AUC of the classification tree model was 0.802 and 0.794, the specificity was 58.29% and 58.82% and the sensitivity was 88.60% and 87.11% in the training and verification sets, respectively (Fig. 3). The AUC difference of the two models in the training and verification sets were not statistically significant (*P* > 0.05). The ROC curve parameters of the two models are shown in Table 3.

**Table 3:** The ROC curve parameters of the two models.

Parameters	Training set			Validation set		
	Nomogram model	Classification tree model	*P* value	Nomogram model	Classification tree model	*P* value
Specificity (%)	63.83	58.29		64.20	58.82	
Sensitivity (%)	84.40	88.60		83.52	87.11	
Youden index (%)	48.23	46.89		47.73	45.93	
AUC (95%CI)	0.806 (0.796 ~ 0.815)	0.802 (0.792 ~ 0.812)	0.1691	0.791 (0.775 ~ 0.806)	0.794 (0.778 ~ 0.809)	0.5537
Cut-off	0.23	0.17		0.22	0.17	

**Figure 4:**
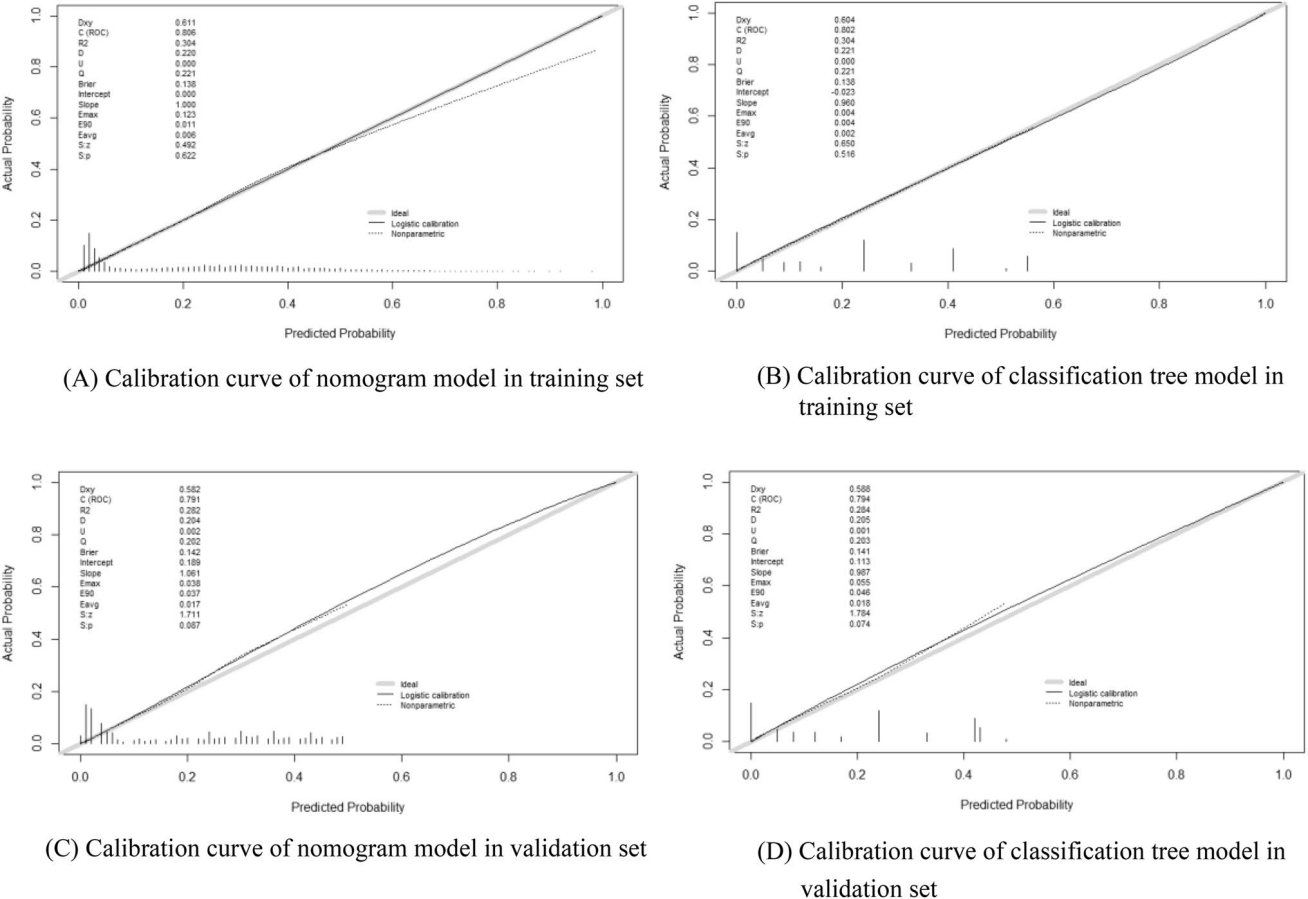
Calibration curves of the two models in the training and validation sets. The dashed line represents the predicted incidence curve, the black solid line represents the actual incidence curve and the gray line represents the ideal reference curve at 45°. *P* > 0.05 in the 4 graphs indicates that the difference between the predicted incidence curve and the actual incidence curve is not statistically significant. The figure was created by using R software (version 4.0.0), URL: https://www.r-project.org/.

In the calibration curve, the predicted incidence curve of the nomogram and the classification tree models in this study fitted well with the optimal curves of the actual incidence, and the difference was not statistically significant (*P* > 0.05; Fig. 4). The results showed that the average absolute error between the predicted risk of HUA occurrence and the actual risk in the training and verification sets of the nomogram model were 0.006 and 0.017, respectively; the average absolute error between the predicted risk of HUA occurrence and the actual risk of HUA occurrence in the training and verification sets of the classification tree model was 0.002 and 0.018, respectively, indicating that the accuracy of the two models was good.

**Figure 5:**
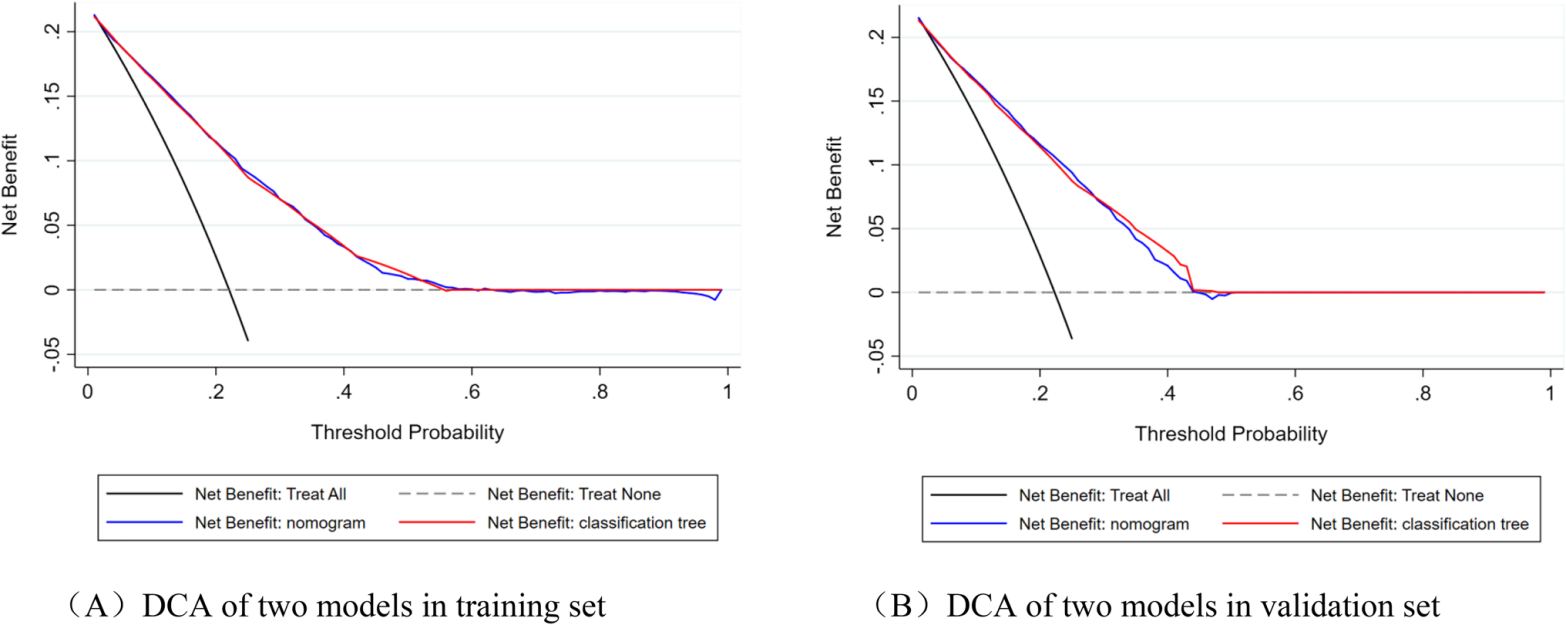
DCA curves of the two models in the training and validation sets. The abscissa represents the threshold probability, and the ordinate represents the net benefit after the benefit (the perceived benefit after treatment for the real patient) minus the disadvantage (the perceived harm after treatment for the fake patient). The dotted reference line indicates that all the samples were free of disease with none of them receiving any intervention, and the net benefit was 0; the black reference line indicates that all of the samples were treated for the disease, and the net benefit is represented by the backslash of the slope. The further the model curve is away from the two reference lines, the better the clinical utility of the graph. The figure was created by using STATA (version 15.1), URL: https://www.stata.com/.

Finally, in order to determine whether the two models had clinical practicality, this study evaluated whether the two models can bring net benefits through DCA. As shown in Fig. 5, in the training and validation sets, both models could bring net benefits within the appropriate threshold probability (Pt) range, and these were deemed to be similar.

## Discussion

The focus of this study was to explore the construction of simple model tools for predicting HUA. We have developed and verified two new and simple HUA prediction models. The nomogram model was selected as a parametric class model, and the classification tree model was selected as a non-parametric class model. We included four non-invasive physical examination indicators that were easy to obtain clinically, such as age, gender, BMI and hypertension, and the results indicated that they were all risk factors of HUA (*P* < 0.05) and were included in the model. The final AUC of the nomogram model obtained was 0.806 and the AUC of the classification tree model was 0.802. The specificity of the nomogram model was higher at 63.68% and the sensitivity of the classification tree model was higher at 88.60%. Each model had its own advantages and disadvantages.

In previous studies, Lee et al.^18^ used gender, BMI and PPARγ genes to establish a model to predict the potential risk of HUA. The final model had a sensitivity of 69.3% and a specificity of 83.7% and although its sensitivity was better than those of the two models in this study, the cost of HUA prediction through genetic testing methods was high, and they were not suitable for rural hospitals with outdated medical equipment and widespread population distribution. Zeng et al*.*^19^ built a HUA prediction model based on dietary information such as the frequency of eating vegetables, meat and eggs, and obtained a neural network model with an AUC of 0.827, a sensitivity of 75% and a specificity of 86%. Its AUC and sensitivity were both better than those of the two models in this study, but the model structure was more complex and it required a higher statistical background knowledge from the operators, and more variables needed inclusion. This model was also time consuming and the subjects were prone to recall bias when it was applied to the clinic.

The nomogram model is based on logistic regression analysis. It is a traditional medical model with a certain amount of accuracy, repeatability, visualization and it does not require computer software intervention. It is often used to explore the risk factors that cause diseases and predict the incidence of diseases through these risk factors. But it is difficult to fully reflect the coupling relationship between variables, and cannot effectively deal with the problem of collinearity^25^. The classification tree model is a non-parametric regression model with no special requirements for the variables included in the model, and continuous variables can also be used as independent variables into the model. It is a type of machine learning model that can effectively deal with the problem of missing independent variables in the data. Not only can the missing values be classified into the category of a mode, but it can also be set as a separate category, so that the result is not affected by the collinearity of the variables. It can also effectively, intuitively and hierarchically display the risk factors and the interactions between them^26^.

In recent years, some scholars have successfully applied the classification tree model to predict myocardial infarction^27^, multi-drug resistant tuberculosis^28^, portal vein thrombosis in patients with acute pancreatitis^29^ and other internal or surgical diseases, and its application in medical-related fields has proven its effectiveness. However, classification trees also have some shortcomings. First, the quantitative explanation of the individual effects of the classification tree on each factor is not as clear as the nomogram model. In this study, logistic regression analysis was used to obtain the OR value, which can clearly determine the specific risk factors that affect the risk of HUA. But the classification tree model can only obtain the importance of ranking for different variables and cannot allocate a specific degree of importance for a particular variable. Second, the classification tree model has poor stability when a small sample size is available. Fortunately, the large amount of data in this study allows the classification tree model to have good stability in its prediction capability.

In this study, the ROC and calibration curves as well as DCA of the nomogram and classification tree models in both the training and validation sets achieved satisfactory results, and they were relatively similar. However, some scholars have used machine learning algorithms such as support vector machines, decision trees and random forests to build HUA prediction models with traditional logistic algorithms. They found that the machine learning model was superior to the logistic model for HUA prediction, which is different from the results of our research. The reasons may be related to the large number of dependent variables in these models and the existence of collinearity^30^. In this study the number of variables included was small and therefore the possibility of collinearity was small, and they were confirmed to be closely related to HUA, so the performance of the nomogram model was relatively stable. In previous studies, it was shown that the prediction accuracy and performance of the nomogram model was better than those of machine learning models such as artificial neural networks and classification tree models^31,32^. Therefore, the quality of a certain model cannot be judged unilaterally, and the accuracy and stability of a single algorithm prediction model are relatively low, which cannot always meet the clinical decision-making needs of multi-sourced, high-dimensional medical data. In future applications, we should pay more attention to the combination of multiple models in order to provide support for the improvement of disease prediction methods.

In this study, the overall prevalence rate of HUA was 21.9% of which 34.9% and 4.1% were seen in male and female populations, respectively. The prevalence rate of HUA in male population was much higher than in females, which agreed with previous reports^33^. However, the lower prevalence of HUA in the female population in this study was considered to be related partly to the latest Chinese guidelines for the diagnosis and treatment of HUA^24^. In this study, the multivariate logistic regression analysis and classification tree model both showed that male gender male accounts for the largest proportion of all risk factors, as was shown previously^34,35^.

The risk of HUA in males is high, and this may be partly due to capacity of estrogen to regulate the levels of uric acid transporter in the kidney through gene expression and thus promoting the excretion of uric acid and reducing its production^36,37^. Therefore, after menopause, the difference in prevalence rates of HUA between males and females gradually decreases^38^. In the classification tree model in this study, the node of female age classification was 49.5 years old, and the prevalence of HUA increased after 49.5 years old, which was roughly consistent with the age of menopausal women. On the other hand, due to work demands and life pressures, men are often associated with unhealthy living habits (such as the consumption of high-purine diets and alcohol), and previous literature has reported that alcohol consumption was a risk factor for HUA^39^. In the nomogram model, the score corresponding to the ages between 18 and 29 years was higher. In the classification tree model, the prevalence of HUA for men younger than 28.5 years was higher than that for men in an older age group, which was the same as that reported by Jin et al*.*^40^. In this study, we found that whether in the training set or the validation set, prevalence of renal insufficiency in the HUA group was lower than that in the non-HUA group, which may be related to the high proportion of men in the HUA group. Previous studies have shown that the prevalence of renal insufficiency in men is lower than that in women, and women is a risk factor for CKD^41–44^. And among HUA patients, the risk of kidney disease progression and CKD in men is significantly lower than in women^45^. However, some studies suggested that the prevalence of renal insufficiency in men is higher than that in women^46,47^. Therefore, the relationship between gender and renal insufficiency needs further studies.

Additionally, the classification tree model suggested that there was an interaction between gender and BMI. For both men and women, the prevalence of HUA in people with high BMI was higher than that in people with low BMI, and this parameter was the second most important influencing factor in the classification tree. Previous epidemiological evidence has shown that obesity was closely related to HUA, and BMI and waist circumference were positively correlated with HUA^48,49^, especially visceral fat obesity, which could accelerate ribose-5-phosphate de novo synthesis of phospho-ribose pyrophosphate through the NADP-NADPH metabolic pathway, and eventually lead to an increase of uric acid and trigger HUA^50,51^. Therefore, the results of this study suggested that obese young men and postmenopausal women are key health management subjects in attempting to prevention HUA.

There are several advantages attributed to this study. First, the predictive variables required in this model were non-invasive and easy to evaluate. In addition to saving medical expenses, limited information could be used to screen undetected and unsuspected high-risk groups of HUA, which ideal for large populations with limited health resources, especially in poorer areas. At the same time, not only China, Africa and India have poor sanitary conditions, they may also benefit from this model. However, it should be borne in mind that some countries define the diagnostic criteria for female HUA as 360 μmol/L. When applying this model, particular attention should be paid as to whether thediagnostic criteria used are the same. In addition, we combined parametric and non-parametric methods to build two prediction models. For practical applications, this method allows the possibility to select different algorithms for comprehensive comparisons and complementary advantages based on the available data characteristics and sample size, so as to improve the reliability of prediction results obtained and expand the scope of application of the model.

However, this study also had several limitations. Our sample size originated from the database of the same medical examination center, and hence there was possible selection bias. Therefore, it may not be possible to determine whether it is applicable to populations in other regions, and further external verification is required. As a cross-sectional survey, this study was unable to confirm the causal and temporal relationships between age, gender, BMI, hypertension and HUA, which needs to be confirmed by a large number of further multi-center, prospective studies. In addition, the model only includes four non-invasive physical examination indicators. Waist circumference, hip circumference and waist-to-hip ratio can also be used as markers of metabolic syndrome as well as certain dietary habits such as high-purine diets. These parameters may be able to improve the performance of the model. In the next step of the study, we will carry out a more extensive study.

## Conclusions

In summary, this study developed and validated two simple HUA prediction models suitable for Chinese and general rural populations by using simple and non-invasive physical examination indexes. The two models have high resolution, good accuracy and some encouraging clinical practicalities, which can potentially help to identify high-risk groups and reduce the prevalence of HUA and gout and the adverse health consequences caused by cardiovascular diseases. In addition, this model can also help save medical resources, reduce the economic burden in poverty-stricken areas, and strengthen the health management of rural community populations. Of course, the model still has some limitations, and repeated clinical studies and external validation in different regions are needed to finally prove whether this model has application and promotion value. In the following research, we can further integrate clinical medicine, epidemiology, biostatistics and other disciplines, and explore whether other variables can be added to improve the performance and application scope of the model, so as to provide some help for the prevention and control of the occurrence and development of chronic diseases.

## Data Availability

The data that support the findings of this study are available on request from the corresponding author. The data are not publicly available due to their containing information that could compromise the privacy of research participants.
